# Pachydermodactyly with Broder Hand Span and Long Fingers: A Case Report

**Published:** 2017-02

**Authors:** Abbas RASI, Elham BEHRANGI, Bahamin ATTAR, Zahra AZIZIAN

**Affiliations:** 1. Dept. of Dermatology, Rasoul-e-Akram Hospital, Iran University of Medical Sciences, Tehran, Iran; 2. Dept. of Orthopaedic Surgery, Shohada Hospital, Faculty of Medicine, Tabriz University of Medical Sciences, Tabriz, Iran; 3. Skin and Stem Cell Research Center, Tehran University of Medical Sciences, Tehran, Iran

**Keywords:** Pachydermodactyly, Digital fibromatosis, Soft tissue swelling

## Abstract

Pachydermodactyly is a rare and benign disease that may be idiopathic, genetic, acquired as a response to repetitive trauma, or associated with several other acquired conditions often pushing the health caregiver to do a bunch of costly lab tests and diagnostic workups to rule out other entities. All health care givers must be aware about this disease for reassure the patients and cut unnecessary costs. Moreover, there seems to be an issue of association with certain occupations. A good example might be computer keyboards causing special damages to certain organs like eyes and musculoskeletal system. We have observed deleterious effects of excess work with computer keyboards on fingers in the form of Pachydermodactyly in our case. A 27-yr-old man presented with wider hand span and longer fingers to Dermatology Clinic of Rasoul-E-Akram Hospital in June 2015, especially the ring finger in our case, considered a big symptom who depressed due to their fingers appearance as a rare disease. We gave him an emollient to make his hand smoother. The patient improved both clinically and psychologically on a simple emollient. This disease with its deleterious psychological effects and a simple way of management should be appreciated more by the health care system.

## Introduction

**Fig. 1:**
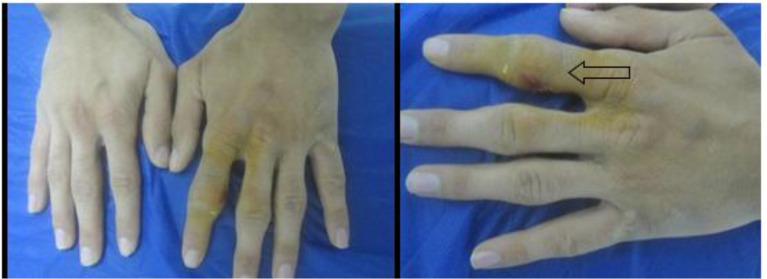
Left Soft-tissue swelling on the proximal interphalangeal joint of the second through fourth fingers with thickening of the skin Right: Close view

**Fig. 2:**
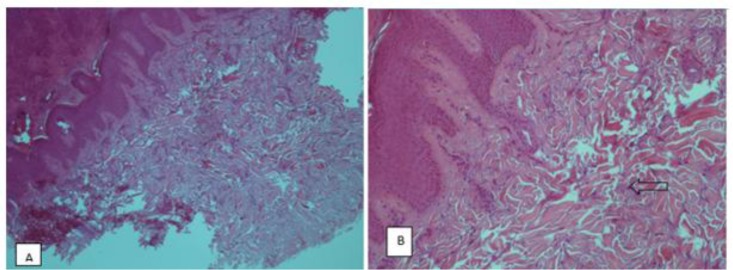
A: Hyper orthokeratosis, acanthosis and thickened dermis (H&E stain, ×10) B: Irregular bundles of thickened collagen (H&E stain, ×40)

Pachydermodactyly (PDD) is a benign superficial fibromatosis of the fingers, characterized by painless bulbous fusiform swellings, affecting the skin and overlying the radial and ulnar aspects of the proximal interphalangeal (PIP) joints (Fig.1–2); affects young adult men (1–2). Here in we present a 27-yr-old male with PDD.

### Case report

A 27-yr-old Iranian man was presented to the Rasuol-Akram Hospital Dermatology Day-Clinic, Tehran, Iran with a 3-yr history of progressive, asymptomatic and fusiform symmetrical swellings, localized to the medial and lateral aspects of several fingers of the left hand. The patient did not have any similar familial history. Furthermore, no history of significant trauma and pain or arthritic symptoms was present. The subject also had relatively long fingers in both hands (right handed, height: 175 cm, weight: 70 kg, hand span: 188 cm and index finger: 105 mm).

Physical examination revealed doughy fusiform swellings, limited to the medial and lateral sides of the PIP joints of the second through to the fifth fingers of the left hand (Fig. 1 a,b). These changes were extended to the metacarpophalangeal (MCP) joints of the second and third fingers of the same hand. These lesions were firm plaques, and overlying skin was lichenified, dry and mildly scaly. The swellings did not cause any range of motion reduction. The patient had no evidence of joint warmth or synovitis. The first and the fifth finger were spared. The X-rays of the affected hand revealed only soft tissue swelling without bone or articular abnormalities or space loss. His left-hand span was broader and his left fingers were longer than right ones, and had borderline joint laxity but without any remarkable signs of Ehlers Danlos. Routine laboratory screening, were normal.

An incisional biopsy was taken through the lichenified skin overlying the left second PIP joint, and stained with hematoxylin and eosin, which revealed marked compact orthokeratosis hyperkeratosis, prominent granular cell layer, mild acanthosis of the epidermis, and thickened, tortuous, haphazardly oriented collagen bundles in the reticular dermis. Normal fibroblasts were evident. No inflammatory infiltrate was present (Fig. 2 a,b). Ehlers Danlos syndrome was ruled out by negative skin biopsy, absence of neurological symptoms and Brighton criteria. Based on the clinical and histologic findings, a diagnosis of PDD was made.

## Discussion

PDDs classified into five major groups: 1) Classic, frequently associated with mechanical trauma, 2) Localized or mono, refers to single digit involvement, 3) Trans gradient, refers to examples of cutaneous thickening extending onto the metacarpophalangeal region, as in our case, 4) Familial, and 5) PDD-associated with tuberous sclerosis (3,4).

The cause of PDD remains unknown (4, 5). Obsessive-compulsive habits and Asperger syndrome may play an important role in PDD etiology (3, 6, 7). However, some authors hypothesized that PDD is probably caused exclusively by emotional stress and consequent repetitive mechanical stimulation such as clapping and flapping of hands quite aggressively or rubbing hands vigorously together (8). The case of this study was a computer workaholic; therefore, friction of the fingers and repetitive mechanical trauma has been suggested as a precipitating factor. However, these associations with wide hand span and long fingers may still be a mere coincidence.

Clinically, PDD is a distinct entity that should be easily differentiated from other causes of cutaneous thickening affecting the hands such as knuckles pads, juvenile fibromatosis, pachydermoperiostosis, and endocrinologic, psoriatic pachyderm dactylies, Juvenile chronic arthritis, and juvenile rheumatoid arthritis (1, 6, 8). PDD is a distinct clinical entity, and a rare form of probably reactive benign digital fibromatosis of unknown etiology (9, 10).

## Conclusion

Rapid recognition of the affected patients is essential for reassuring patients and preventing unnecessary broad investigations and treatments.

## Ethical considerations

Ethical issues (Including plagiarism, informed consent, misconduct, data fabrication and/or falsification, double publication and/or submission, redundancy, etc.) have been completely observed by the authors.
